# Oral health-related quality of life of Portuguese adults with mild intellectual disabilities

**DOI:** 10.1371/journal.pone.0193953

**Published:** 2018-03-21

**Authors:** Patrícia Couto, Paulo Almeida Pereira, Manuel Nunes, Rui Amaral Mendes

**Affiliations:** 1 Faculty of Health Sciences, Beira Interior University, Covilhã, Portugal; 2 Department of Economics, Management and Social Sciences, Portuguese Catholic University, Viseu, Portugal; 3 Department of Oral and Maxillofacial Medicine and Diagnostic Sciences, School of Dental Medicine, Case Western Reserve University, Cleveland, Ohio, United States of America; Navodaya Dental College and Hospital, INDIA

## Abstract

Individuals with disabilities are regarded as a highly vulnerable population group, particularly as far as oral health is concern. However, few studies have assessed the impact of the oral condition on the quality of life of these individuals. Therefore, the aim of this study is to expand knowledge on the oral health status of the Portuguese adults with mild intellectual disability, and to assess how the patient’s oral health is related to their quality of life. A sample of 240 adults with mild intellectual disabilities linked to the Portuguese Federation for Intellectual Disability, were interviewed using a previously validated version of the Oral Health Impact Profile. An oral health examination was also conducted using three oral health indexes: Clinical Oral Health Index (COHI); Clinical Oral Care Needs Index (COCNI) and the Clinical Oral Prevention Index (COPI). Sociodemographic characteristics and dental health factors were also collected, following statistical analysis. More than half of the individuals (54,9%) presented one or more problems of major to severe impact on health (COHI level 2); only 4,6% of the individuals do not need treatment or examination (COCNI level 0) and 85% of the study sample needs measures of educational or preventive action (COPI level 1). In 76,9% of the participants, oral health had impact on the quality of life. The most affected dimensions of life were physical pain with 61,9%, followed by psychological discomfort and psychological disability with 45,1% and 45%, respectively. With relation to oral health factors and sociodemographic variables it was verified that fewer teeth and higher self-perception of need for dental treatment had a negative impact on the quality of life. On the other hand, institutionalization and an increase in at least one category in the self-perception of the oral health status had a positive impact on the quality of life. Given the high burden of oral disease and the considerable impact on quality of life found in this study, the establishment of guidelines to improve the oral health and quality of life of these individuals should be regarded as imperative.

## Introduction

Experiencing inequality in health services is, unfortunately, a reality for people with disabilities, with disparities being found in many health sectors, including oral health [1–5].

Dental care among adults with intellectual disability is one of the most unattended health needs. Several studies evidentiate that people with intellectual disabilities have worse oral hygiene, increased tooth decay and also a worse periodontal condition, compared to people with no disabilities [6–11]. This situation is often negatively influenced by different factors: intrinsic limitations, poor general health, polymedication, low socioeconomic level, type of residence, degree of family and caregiver’s commitment, social barriers, barriers related to the health professional and political barriers [4,6,12,13].

When oral health care does not meet the needs of the individuals, it can negatively impact their general health and wellbeing, deteriorating quality of life [14], as a weak oral health status may cause, among others, pain, sleep disturbance, decreased self-esteem, discomfort and an unsatisfactory diet [7,15].

Assessing only clinical signs, without exploring how people perceive their oral health and the impact it has on their quality of life cannot describe the people’s subjective perceptions, satisfaction, self-esteem or the ability to perform daily activities. A better notion can be obtained looking not only to the oral health status diagnosed on the clinical exam but also to the subjective points related with oral health. In this way, oral health-related quality of life (OHRQoL) indicators can be a great help [16], as they allow us to assess how oral health or disease affects people’s daily life and well-being [17].

The oral health impact profile questionnaire (OHIP-14), which is composed by 7 dimensions based on 2 questions each, has been widely used in several countries, and, according to Cummins [18], most people with intellectual disabilities can respond in a reliable form to subjective questionnaires about their quality of life of which they have a clear perception.

Nonetheless, the assessment of oral health care needs and, specially, the impact of oral health in the quality of life of people with intellectual disability, has been somehow disregarded, despite the fact that life expectancy of people with intellectual disability has followed similar trends to those found in the general population, therefore making adulthood, which were not recognized in this population, an important reality of these patients’ life course [19]. Thus, the current study aims to understand the oral health condition of a cohort of patients with mild intellectual disabilities and associated risk factors, therefore contributing to a better understanding of how oral health might be properly addressed as part of an holistic approach of this patients’ health problems, hence positively influencing their life expectancy.

In addition, we sort to investigate the association between self-reported and clinical oral health status and assess the prevalence of the negative functional and psychosocial outcomes of oral disorders on the quality of life of adults with mild intellectual disability.

## Materials and methods

### Study design and setting

This research consists of an epidemiological, observational cross-sectional study on oral health and quality of life of people with mild intellectual disability, linked to the intellectual disability institutions of the Central Region of Portugal affiliated to Humanitas (Portuguese Federation for Intellectual Disability) and developed over the course of 2016. First, a modified version of the OHIP-14 questionnaire was validated for the population under study—mild intellectual disabilities/ Portugal -, which we called OHIP-14-MID-PT. Then, a questionnaire to collect sociodemographic and self-reported oral health data was used with the previously mentioned version of the OHIP-14 questionnaire. Finally, a clinical examination was performed based on the clinical oral health index (COHI), the clinical oral care needs index (COCNI) and the clinical oral prevention index (COPI) [3]. In order to complete the questionnaires and to perform the oral examination, written informed consent was previously distributed and orally explained to all participants and always in the presence of family members/caregivers. Participation in the study was voluntary, free and unpaid. The Ethics Committee of the Faculty of Health Sciences of the University of Beira Interior and the Ethics Committee of APPACDM—Viseu; APPACDM -Coimbra; APPACDM—Figueira da Foz; APPACDM Vila Nova de Poiares and Arcil- Lousã approved this research, which was performed according to the principles of the Helsinki Declaration, version 2013.

### Participants

The 13 institutions affiliated to Humanitas and located in the Central Region of Portugal provided care to 556 individuals with mild intellectual disabilities. After obtaining the agreement of the institutions in participating in this study, the sample was calculated for an error estimate of 5%, resulting in a minimum sample size of 228 individuals. The power calculation based on sample size was 79,5%. In order to avoid possible bias errors (no responses, difficulties in collecting clinical data), a significantly higher margin, 288 (around 25%), was given to ensure at least a minimum sampling value. Of these, 240 met the inclusion criteria to participate in the present study.

In order to overcome illiteracy problems and avoid any situation that might impair the filling, the questionnaire was used, as such, in the course of an interview by a pre-trained researcher.

Exclusion criteria were: non-cooperating users whose behavior or medical condition makes the clinical examination impossible, aged under 18 years, subjects who do not consent to participate through informed consent, and subjects who were absent on the day of the examination.

### Data collection instruments

Data from the individuals was collected through clinical examination guided by the Clinical Oral Health Index (COHI), Clinical Oral Care Needs Index (COCNI) and Clinical Oral Prevention Index (COPI) [3] S1 File and through interviewer-administered questionnaires by a single dentist (OHIP-14-MID-PT S2 File and sociodemographic/oral health questionnaires). The questionnaires were pre-tested prior to its application and the intra-examiner reliability was evaluated by reassess of 20 individuals with an interval period of two weeks.

#### Sociodemographic and oral health questionnaire

The World Health Organization provides questionnaires to collect data of self-perception of oral health, such as the Oral Health Questionnaire for Adults, which has already been tested in pilot studies in numerous countries and which was taken into account in the structuring of this study’s oral health questionnaire.

Socio-demographic variables include: gender, age, residence, institution, location, years and type of relationship with the institution.

Oral health variables include: number of natural teeth, self-perception of oral health and oral health care needs, use of dentures, frequency and hygiene methods, frequency and reason for going to the dentist, smoking, alcoholic and food habits.

The subjective impact of oral conditions was determined by two questions. One about self-perceived need for dental treatment, using response categories as "Yes", "No" and "I do not know", and another about self-perception of oral health status, categorizing it on an ordinal scale as "Excellent", "Very good", "Good", "Medium", "Weak", "Very weak" and "I do not know".

#### Questionnaire OHIP-14-MID-PT

OHIP-14-MID-PT was used for assessing OHRQoL and it consists of 14 questions, with 7 dimensions (pain, functional limitation, psychological discomfort, physical disability, psychological disability, social disability and handicap) of 2 questions each.

Each question was rated on a 5-point Likert scale (0- never; 1- hardly ever; 2- occasionally; 3- fairly often; 4- very often). Total possible scores ranged from 0 to 56, with higher scores indicating poorer quality of life. Prevalence of the oral health impact profile was quantified as the proportion of adults who reported experiencing one or more impacts, occasionally, fairly often or very often within the past year.

#### Clinical examination

The clinical examination criteria were adapted from three original clinical indexes generated from an algorithmic association of several clinical indicators which allows an adequate measurement of the oral conditions in subjects with disability. The COHI consists of 4 levels, assuming the values of 0, 1, 2 or indeterminate, depending on the oral health problems of the individual. The COCNI, made up of 4 levels (0, 1, 2, 3), allows to assess and determine the dental needs of each individual. The COPI, in turn, determines possible needs in terms of dental education initiatives. In this way, it will assume level 0 when there is no need for preventive and oral health education actions, and level 1 when there is a need for at least one prevention or education action [3].

The clinical examination was carried out in the premises of each institution, in a medical room provided for this purpose. Users were seated in chairs with armrests, with sufficient headrest and in a lower position than the examiner, taking advantage of the natural luminosity and the artificial one from the lamps, in order to obtain adequate visibility and positioning.

Nurses from each institution, as well as other medical assistants were present, assisting the examiner in communication and controlling behaviors when necessary. The materials used for the observation were all of single use: mirrors, compresses, exploratory and periodontal probes that comply with WHO specifications, tweezers, gloves, protective mask, white coat, disinfectant solution and a front lamp.

The medical record of each patient was analyzed prior to the examination and all data was kept confidential.

The results of intraoral examinations were reported to the institution's management and to the users. The information collected was used for the subsequent provision of dental services.

The principles of cross-infection control, using material that was all disposable, sterilized and opened only at the time of clinical examination, were taken into account.

### Data analysis

For the data collected through the interviews, simple frequency distributions highlighted the key themes and these are also presented narratively in the Results chapter.

Non-parametric test of Kruskall-Wallis, non-parametric test of Mann-Whitney, Pearson's Correlation Coefficient and Chi Square test were used for comparison of OHIP-14-MID-PT scores with the oral health/sociodemographic variables and the results of the clinical examination.

Binary logistic regression analysis was adopted to explore the relationship between oral variables and OHIP-14-MID-PT score, and in that way find risk predictors for oral disorders. Binary logistic regression was applied, using the Hosmer-Lemeshow statistic to determine the quality of fit, being a non-significant result indicative of good fit quality.

The Omnibus test can be interpreted as a test of the ability of all predictors in the model to estimate the variable (dependent) response, where a significant test value (less than 5%) corresponds to a conclusion that there is adequate adjustment of the data to the model.

The coefficient of determination R2 can not be calculated for binary logistic regression models, so we use an approximation of pseudo R2 calculation: R2 of Nagelkerke's.

In binary logistic regression, the highest category (1 = impact) is estimated and the lowest category (0 = no impact) is the benchmark comparison. If the test value is less than 5% (0,05), then the associated independent variable is significant for the model.

Statistical analyses were performed using SPSS version 22.0 and a 0,05 significance level.

## Results

### Descriptive analysis of OHIP-14-MID-PT

Analyzing the results of OHIP-14-MID-PT, there was an impact on quality of life of 9,98 ± 10,79, with 76,9% of the sample having some type of impact. The most affected dimensions were physical pain with 61,9%, followed by psychological discomfort and psychological disability with 45,1% and 45%, respectively. While the least affected dimensions were social disability with 22,6% and handicap with 19,7%.

### Clinical examination and OHIP-14-MID-PT results

**Fig 1:**
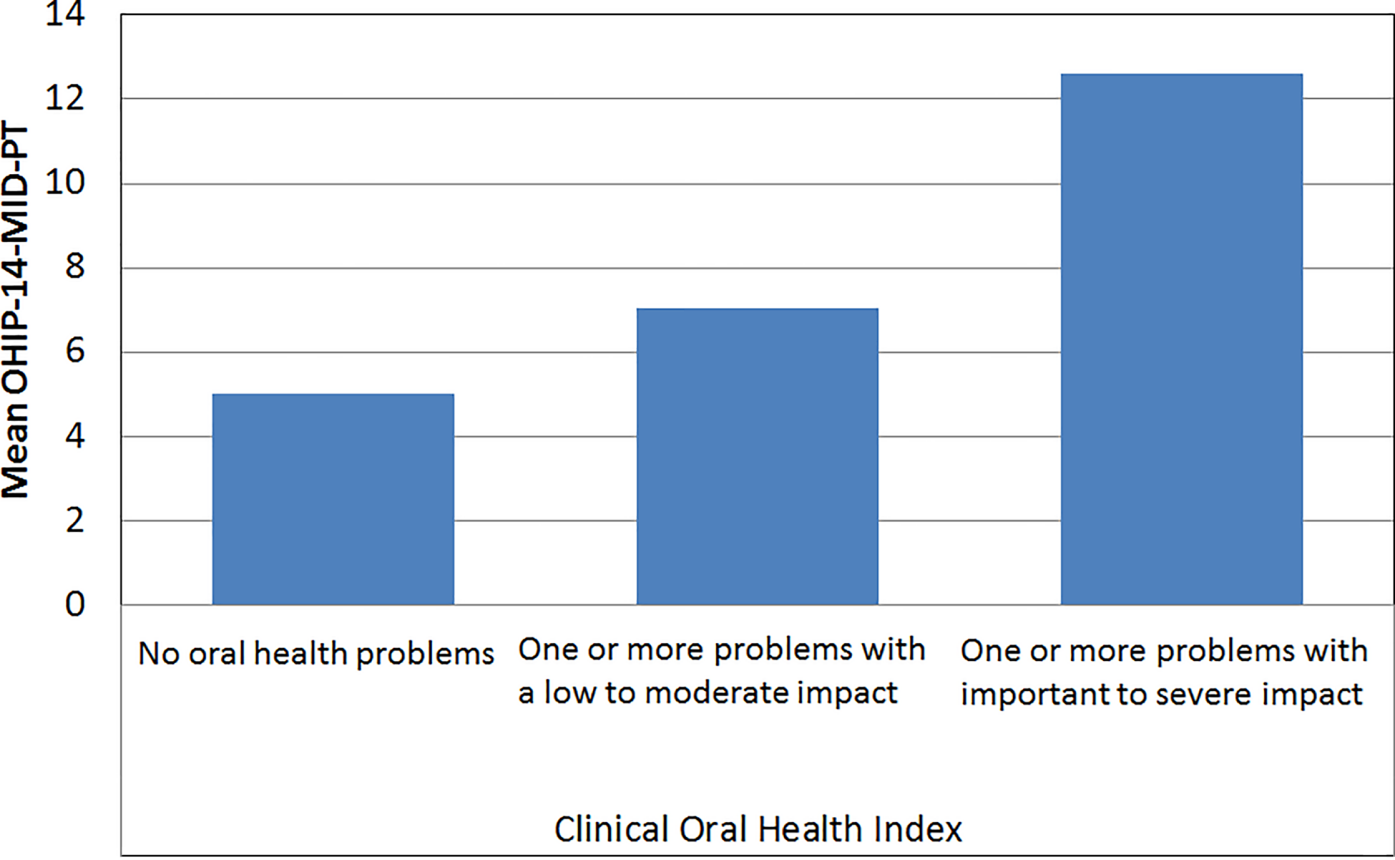
Relationships between OHIP-14-MID-PT and the Clinical Oral Health Index.

More than half of the sample (54,9%) presented one or more problems of major to severe impact—COHI level 2 and only 2,1% presented no oral problems—COHI level 0. The quality of life was perceived through the scale OHIP-14-MID-PT as higher among those who had COHI level 0 compared to those who had COHI level 1 or 2. (χ2 = 18,50; p<0,001) (Fig 1).

**Fig 2:**
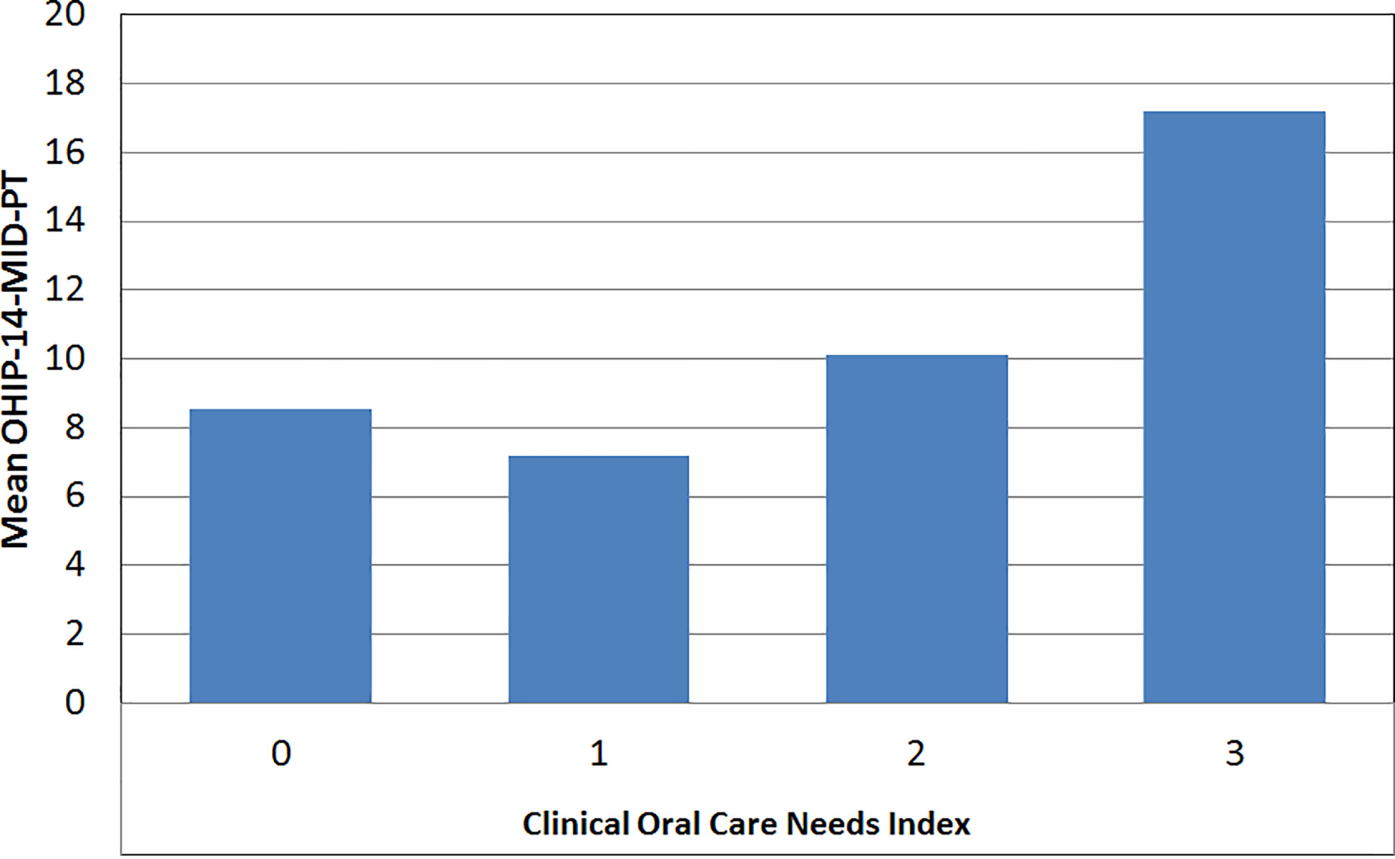
Relationships between OHIP-14-MID-PT and the Clinical Oral Care Needs Index.

In relation to treatment needs, 4,6% of the individuals do not needed treatment or examination (COCNI level 0), 26,3% required examination (COCNI level 1), 58,8% needed care or examination (COCNI level 2) and 10,4% needed urgent care/examination (COCNI level 3). Quality of life was perceived as lower for those who had COCNI level 3, followed by those who had COCNI level 2 (χ ^2^ = 16,37; p = 0,001) (Fig 2).

**Fig 3:**
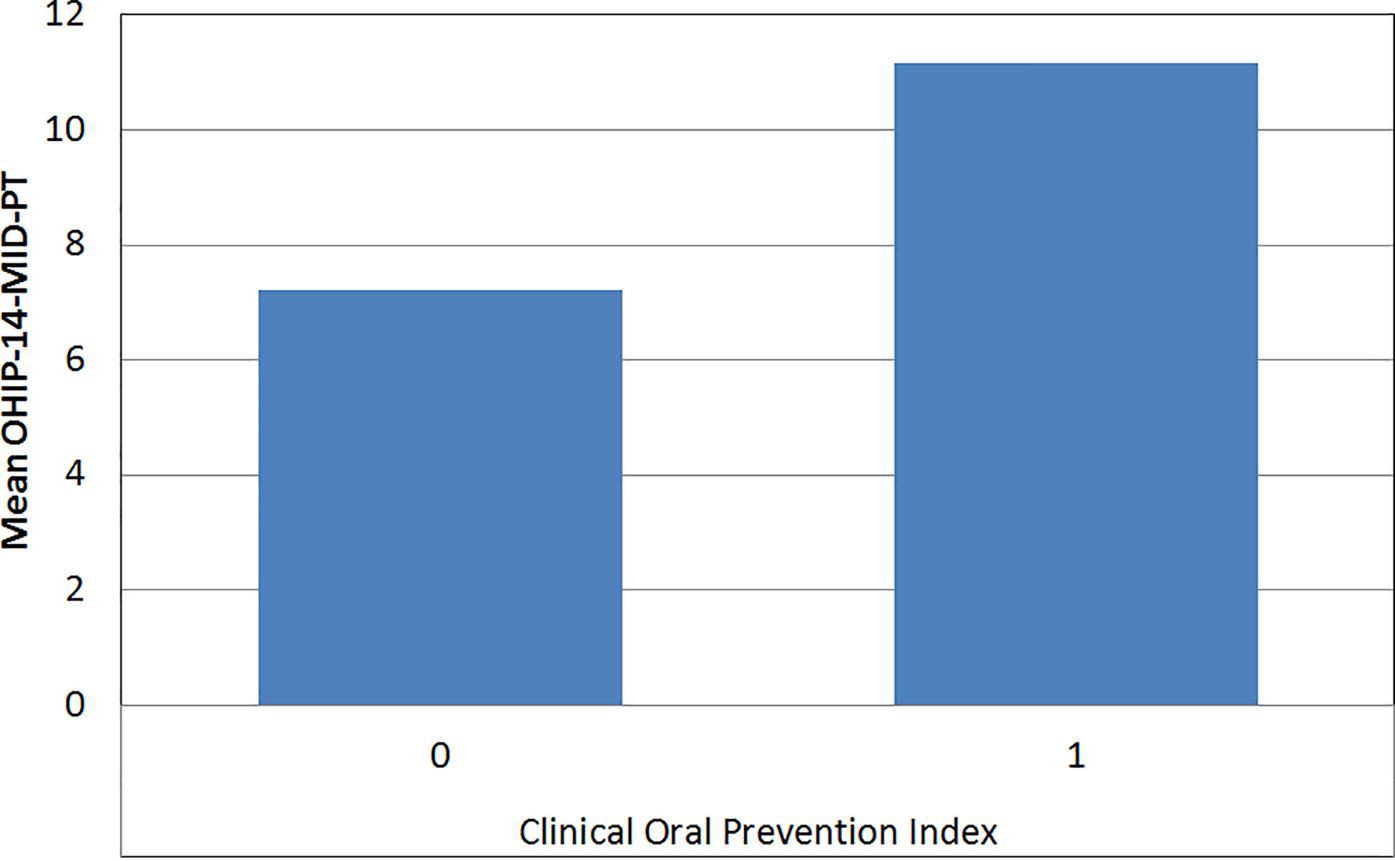
Relationships between OHIP-14-MID-PT and the Clinical Oral Prevention Index.

In the present sample, 85% of the individuals needed at least one measure of educational or preventive action (COPI level 1). In the global OHIP-14-MID-PT scale there were no statistically significant differences (U = 2597,5; p = 0,135) among those with COPI level 0 and those with COPI level 1. (Fig 3).

### Oral health status and oral hygiene–objective and subjective analysis

Of the 240 subjects participating in the study, 79 (32,9%) had fewer than 20 teeth and 15% presented prosthetic rehabilitation.

The quality of life was perceived as higher among individuals with 20 or more teeth compared to those with less than 20 teeth, however, those who do not had any teeth had an average value of self-perception of quality of life similar to those with 20 teeth or more (χ^2^ = 29,74; p<0,001), possibly due to prosthetic rehabilitation.

Furthermore, 79,6% of the respondents stated daily oral hygiene; 18,8% brush only occasionally, and 1,7% affirmed never brush their teeth or dentures. In the sample, the percentage of individuals with COHI level 2, COCNI level 3 and COPI level 1 on clinical examination was higher for those who never brush their teeth and it was also verified that those who used dental floss presented better results at the intra-oral clinical examination (χ^2^ = 9,66; p = 0,008) and consequently lower dental care needs (χ^2^ = 8,86; p = 0,031) and lower needs for preventive measures (U = 3258,0; p = 0,015) than those who did not use it.

It should also be noted that 7,2% of the sample never went to the dentist and only 28,4% had a dental appointment in the last 6 months. Only 18,8% referred going to the dentist for routine dental appointment, and younger individuals were more likely to seek the dentist on a routine basis or for medical advice. (χ^2^ = 7,51; p = 0,023). In the sample, the percentage of COHI level 2 and COCNI level 3 was higher for those who have not been to the dentist for more than 5 years than those who went to the dentist less than 12 months ago.

### Self-perception of need for dental treatment and self-perception of oral health status

Of the 226 individuals who answered the question “Do you feel that you need any type of dental treatment?” of the health questionnaire, 170 (75,2%) considered needing some kind of treatment. The quality of life was perceived as higher in the global OHIP-14-MID-PT scale by those who also did not feel they needed some type of dental treatment, vide Table 1.

**Table 1:** Descriptive statistics and Mann-Whitney tests. Relations between OHIP-14-MID-PT and the question “Do you feel that you need any type of dental treatment?”.

	Q7	N	Mean	SD	U Mann-Whitney	P
OHIP-14-MID-PT	No	52	5,37	7,657	2366,5	** 0,000
	Yes	156	11,89	11,497		
1. Functional Limitation	No	56	1,00	1,868	3920,5	0,059
	Yes	165	1,35	1,756		
2. Physical Pain	No	55	1,51	1,875	3127,5	** 0,000
	Yes	170	2,68	2,080		
3. Psychological Discomfort	No	55	,76	1,440	2865,5	** 0,000
	Yes	166	2,25	2,345		
4. Physical Disability	No	56	1,09	1,751	3868,5	* 0,027
	Yes	169	1,83	2,206		
5. Psychological Disability	No	55	,45	1,015	2497,5	** 0,000
	Yes	169	2,23	2,255		
6. Social Disability	No	55	,49	,979	4153,5	0,181
	Yes	167	1,08	1,948		
7. Handicap	No	56	,29	,803	3772	** 0,005
	Yes	168	1,02	1,766		

* significant for p < 0,05

** significant for p < 0,01

There was also an agreement between the treatment needs perceived by the individual and the results obtained in the clinical examination.

A statistically significant negative correlation (r = -0,545, p<0,001) was verified between the OHIP-14-MID-PT scale and the question “How would you describe the condition of your teeth and gums?” which means that individuals who had a positive self-perception of the state of their teeth and gums presented lower OHIP results and vice versa.

### Institutionalized and non-institutionalized individuals

Of the total sample studied, 13,3% were institutionalized. On the OHIP-14-MID-PT global scale, quality of life was perceived as higher among the institutionalized ones (U = 1955,5; p = 0,010). Actually, the percentage of COHI level 1 was higher for institutionalized subjects and level 2 for non-institutionalized. However, the differences were not statistically significant (χ^2^_(2)_ = 3,743; p = 0,154).

For non-institutionalized individuals, the quality of life was perceived as higher by those who live with parents and other relatives and lower by those who live alone (χ^2^ = 22,39;p<0,001). At the clinical examination, the percentage of problems of significant to severe impact was more meaningful for those who live alone, while the absence of problems occurred more often for those who live in the home of friends/host families (χ^2^_(8)_ = 28,387;p<0,001). The same happens with the treatment needs (χ^2^_(12)_ = 30,181;p = 0,003).

### Logistic regression model: OHIP-14-MID-PT and significantly related variables

The statistically significant independent variables related to the OHIP-14-MID-PT scale are shown in Table 2. The OHIP-14-MID-PT scale is the dependent variable. For this model, there are 172 valid cases, corresponding to 71,7% of the sample, due to the existence of 68 cases with *missing values*.

**Table 2:** Estimation of the parameters for the dependent variable OHIP14 impact (reference category: No impact).

						Odds Ratio		
							95% CI for Exp(b)	
	b_i_	s(b_i_)	Wald	Df	p	Exp(b)	Lower	Upper
Location	,009	,561	,000	1	0,987	1,009	,336	3,030
1. Gender	-,673	,567	1,411	1	0,235	,510	,168	1,549
3. Years of relationship with the institution	-,022	,025	,735	1	0,391	,979	,931	1,028
4. Type of relationship with the institution	-1,389	,704	3,887	1	* 0,049	,249	,063	,992
Q6. 20 or more teeth (reference)			5,832	2	0,054			
Q6. 1–9 teeth	3,424	1,637	4,374	1	* 0,036	30,687	1,240	759,388
Q6. 10–19 teeth	2,478	1,185	4,375	1	* 0,036	11,919	1,169	121,544
7. Self-perception of oral health care needs	1,373	,648	4,489	1	* 0,034	3,946	1,108	14,049
8. Use of dentures	-1,258	1,358	,858	1	0,354	,284	,020	4,070
9. Self-perception of oral health	-,755	,338	4,986	1	* 0,026	,470	,242	,912
Fresh fruit	-,091	,182	,249	1	0,618	,913	,640	1,304
Biscuits and cakes	-,023	,218	,011	1	0,915	,977	,637	1,498
Jellies or honey	,030	,187	,026	1	0,873	1,030	,714	1,488
Chewing gum	-,124	,200	,388	1	0,533	,883	,597	1,306
Sweets	,428	,236	3,297	1	0,069	1,534	,967	2,434
Soft drinks	,111	,195	,325	1	0,569	1,118	,762	1,639
Tea with sugar	,003	,149	,000	1	0,982	1,003	,750	1,342
Coffee with sugar	,042	,138	,091	1	0,762	1,043	,795	1,367
15. Smoking habits	-,284	,713	,159	1	0,691	,753	,186	3,047
Clinical Oral Health Index	,097	,622	,025	1	0,875	1,102	,326	3,728
Clinical Oral Needs Index	,123	,537	,053	1	0,818	1,131	,395	3,242
Constant	2,309	2,081	1,231	1	0,267	10,060		

* significant for p < 0,05

#### Model fit tests

To validate the model, two fit tests were conducted: in the Hosmer-Lemeshow Test, the p value is higher than 5% (p = 0,879), so, the model fits the data properly. In the Omnibus Test to the coefficients of the model, the p value is 0,0%, less than 5% so, we may conclude that the model fits adequately the data, in terms of the existence of variables with predictive capacity. The Pseudo R^2^ that indicates the variation of the dependent variable explained by the model was also determined and the value of Nagelkerke's R^2^ is 45,8%.

In the Table 2 the results of the regression are shown: parameters coefficients b for the independent variables, respective standard error, Wald statistic and their significance, and the interpretable odds ratio value Exp(b).

The probability of impact on the OHIP-14-MID-PT scale decreases by a factor of 0,249 for the institutionalized people and decreases by a factor of 0,470 for an increase of one category in the self-description of the oral health status. The probability of impact on OHIP-14-MID-PT increases by a factor of 30,687 for those with 1–9 teeth compared to those with 20 teeth or more; increases by a factor of 11,919 for those who have 10–19 teeth compared to those who have 20 teeth or more and increases by a factor of 3,946 for those who feel the need for dental treatment.

Of the total cases, 83,1% are correctly estimated from the model, however, it should be noted that only 47,2% of the non-impact cases are correctly estimated, vide Table 3.

**Table 3:** Practical results of using the model.

	Estimated		
	OHIP14Impact		Correct
OHIP14Impact	Without impact	With impact	Percentage
Without impact	17	19	47,2
With impact	10	126	92,6
			83,1

## Discussion

This study revealed that the oral health status of people with mild intellectual disability has a huge impact on the quality of life, with 9,98 ± 10,79 (76,9%) of the sample suffering from some type of impact. Similar results (80,7%) were obtained by the Spanish version of OHIP-14sp [20] for the adult population. Other studies, involving more extensive adult populations without intellectual disability and using the same form of dichotomization of responses, had prevalence of one or more impacts in 50,3% [21] and 51% [22] of the individuals.

The results demonstrated that physical pain, psychological discomfort and psychological disability are the most affected dimensions, followed by physical disability, functional limitation and, finally, social disability and handicap, which confirms that the problems are not only a source of pain, but also a cause of physical and emotional illness [23]. These results resemble those of Nuttal et al. [22] which obtained physical pain (40%) as the most affected dimension, followed by psychological discomfort (27%), psychological disability (18%) and, finally, handicap (8%) and social disability (8%) as the less affected dimensions; and those of Montero-Martín et al. [20] which identified as the most affected dimensions psychological discomfort (53,7%), functional limitation (51,1%) and physical pain (42,2%), followed by social disability and handicap as the less affected dimensions.

However, our results differ from other studies, in which psychological discomfort overlaps physical pain and in which the least reported problems are physical disability and functional limitation [21]. These differences may be due to the differences in the conceptualization and interpretation of questions, differences in population characteristics and/or differences in the perception of oral health severity among different populations, as already verified in other validations [21,24].

Information about the objective and subjective oral health needs of people with disabilities is fundamental to break down barriers and create a greater access to oral health care and better oral health conditions [25].

With regard to the objective oral health exam performed, we found that 54,9% of the sample presented one or more problems of major to severe impact (COHI level 2) and only 2.1% presented no oral problems (COHI level 0), similarly to the study developed by Hennequin [3] that found 49% of COHI level 2 and 6,3% of COHI level 0 in a sample group of children with disabilities and 47,8% of COHI level 2 and 1,8% of COHI level 0 in a sample of adolescents with disabilities attending special schools in France. Other studies about various disabilities show a high prevalence of dental caries and a huge need for restorative and prosthetic dental care [25–27].

These results are even more worrisome if we think that the data may be regarded as potentially underestimated, since we did not perform any x-rays during our clinical examination [25]. In relation to the dental needs, the present study showed that 4,6% of the individuals did not need care or examination (COCNI 0), 26,3% required examination (COCNI 1), 58,8% needed care (COCNI 2) and 10,4% needed urgent care/examination (COCNI 3). These results present relatively similar values to those of the study leading to the creation and validation of the indexes [3] where 9,8% of children with disability did not need care or examination; 31,6% required examination; 40,7% needed care and 17,8% needed urgent care or examination. Of the adolescents with disabilities, 8,2% showed no need for care or examination; 23,5% needed examination; 50,6% needed care and 17,7% needed urgent care/examination.

The Clinical Oral Prevention Index score was often 1 which demonstrates the high needs in terms of dental education initiatives. Using the COPI, Hennequin and her research team [3] demonstrated that more children with disability were in need of preventive oral health care and oral health education (41,3%) than children without disability (21,3%) and that adolescents and young adults with disabilities (51,9%) had greater needs for preventive oral health care or oral health education actions than children. Our results were higher, possibly due to the fact that older adults have more systemic diseases requiring specific oral health monitoring, which is a criterion for the COPI to assume the value 1.

Other researches demonstrate a poor level of oral hygiene, and inadequate scores of dental plaque and calculus accumulation in people with some kind of disabilities [25,26], emphasizing the need for preventive measures.

In our study, we also verified a considerably low number of natural teeth, probably due to a deficient provision of oral health care (causing teeth affected by dental caries to be extracted rather than treated [28]), or/and due to the high prevalence of periodontal disease (associated with genetic impairment and also poor oral health care) in people with intellectual disabilities [29]. Moreover, the presence of insufficient teeth leads to changes in eating habits (poor nutrition) with repercussions on the general health of the individual [30]. We also found that only 15% of the sample had dentures, maybe due to the barriers (intrinsic and socioeconomic), which lead to an highly impaired oral rehabilitation of these patients with dental prosthesis, added by other concerns such as the risk of swallowing or the difficult to actually tolerate the use of a denture [25].

Our study illustrates that the likelihood of having an impact on OHIP-14-MID-PT increases for those with 1–9 and 10–19 teeth and for those who require dental treatment and decreases for institutionalized patients and for those with better perception of teeth status. The results obtained in the present study are similar to those disclosed in other quality of life studies in elderly people which frequently reports the association between poor OHRQoL and the self-perception of dental treatment needs and fewer number of teeth [31].

In our study, the prevalence of oral diseases, the need of oral treatments and oral health prevention in institutionalized individuals with mild intellectual disability are lower than in individuals living on their own, or living with relatives and integrated in the society. The quality of life on the OHIP-14-MID-PT global scale, is also perceived as higher by the institutionalized ones, which confirms that caregivers play an important role in the oral health status of disabled people [10,17]. Other studies also show correlation between deinstitutionalization and poor oral health and access to health care [32]. Deinstitutionalization leaves people without both proper assistance and access to dental care services. Thus, our findings show a positive correlation between greater independence and worse oral health, as well as an increase in oral disorders, possibly due to the absence of trained caregivers and less orientation and control in carrying out daily oral hygiene [32].

Unlike what happens in other studies [33], we can consider the combination of a subjective assessment with the objective information of a clinical examination to be one of this study’s strengths. As limitations, we emphasize that: 1) the study is limited to people with mild intellectual disabilities; 2) the lack of studies in Portugal that allow comparisons with other quality of life instruments in this population; 3) and the lack of a control group. Despite these, we decided to compare, whenever possible, the current results with the recently gathered data in the general population, as well as other studies [25].

In summary, we believe that the OHIP-14-MID-PT might be regarded as a useful tool in terms of understanding the overall impact of intellectual disabled patients’ self-perception of their oral health status in their daily activities and quality of life. Furthermore, since we found a high burden of oral disease with a substantial impact in quality of life, especially in non-institutionalized patients, we believe our results support that this may be regarded as a most-valuable tool, which can be used to extract epidemiological data that may help to support the development and implementation of Public Health policies tailored to address the specificities of these patients.

## Supporting information

S1 File Clinical Oral Health Index (COHI); Clinical Oral Care Needs Index (COCNI) and Clinical Oral Prevention Index (COPI). (PDF)

S2 File OHIP-14-MID-PT (Portuguese version of the Oral Health Impact Profile adapted to people with mild intellectual disabilities). (PDF)
