# Supplementary material for: Oral health-related quality of life of Portuguese adults with mild intellectual disabilities
Source: PLoS One. 2018 Mar 21;13(3):e0193953. doi: 10.1371/journal.pone.0193953 (PMC5862473; doi:10.1371/journal.pone.0193953)
Supplement: S1 File — (PDF) [file pone.0193953.s001.pdf]

Índices Clínicos COHI; COCNI e COPI

COHI Values			CRITERIA
**0**			**Having no criteria that had a medical impact:**
No oral health problems			No mucosal lesion on lips, tongue or jaw
		and	Absence of dental plaque (Greene and Vermillon index = 0 for both arches) [35]
		and	Absence of calculus (Greene and Vermillon index = 0 for both arches) [35]
		and	No gingivitis (Loe and Silness index = 0 for both arches) [36]
		and	No fractured anterior tooth
		and	No missing anterior tooth
		and	No missing posterior tooth
		and	No dental caries
		and	No infectious disease
		and	No severe orofacial dysmorphology
			**AND having no criteria that had a social impact:**
			No halitosis
		and	No drooling
		and	No anterior tooth with a fractured, unrestored crown
1	**OR**		**Having no criteria that had a medical impact:**
Existence of one or more problems with a low to a moderate impact on health			No mucosal lesion on lips, tongue or jaw
		or	Absence of dental plaque (Green and Vermillon index = 0 for both arches) [35]
		or	Absence of calculus (Green and Vermillon index = 0 for both arches) [35]
		or	No gingivitis (Loe and Silness index = 0 for both arches) [36]
		or	No fractured anterior tooth
		or	No missing anterior tooth
		or	No missing posterior tooth
		or	No dental caries
		or	No infectious disease
		or	No orofacial dysmorphology
			**AND having at least one criteria that had a social impact:**
			Presence of halitosis
		or	Presence of drooling
		or	At least one anterior tooth with a fractured unrestored crown
			**Having at least one criterion that had a medical impact**
			At least one mucosal lesion on lips, tongue or jaw
		or	Dental plaque (Greene and Vermillon index >0, on a group of teeth or all the teeth of at least one arch) [35]
		or	Calculus (Greene and Vermillon index >0, on a group of teeth or all the teeth of at least one arch) [35]
		or	Localized gingivitis (Loe and Silness index >0 on a group of teeth or at least one arch) [36]
		or	At least one anterior tooth with a fractured, unrestored crown
		or	One limited anterior edentulous segment (1 or 2 anterior teeth)
		or	At least one minor posterior edentulous segment (missing all molars and premolars on 1 or 2 half-arches with at least one residual inter-arch dental contact)
		or	At least one incipient carious lesion, but no developed carious lesion (stage 1 or 2 according to the Eckstrand classification for carious lesions) [37]
		or	Presence of a simple orofacial dysmorphology
			**... regardless of the criteria that had a social impact**
2			**Having at least one criteria that had an important medical impact**
Existence of one or more problems with important to severe impact on health			Generalized gingivitis (Loe and Silness index >0 for both arches) [35]
		or	Missing at least 3 anterior teeth
		or	At least one major posterior edentulous segment (missing all molar and premolars on at least 2 half-arches, without any residual inter-arch dental contact)
		or	At least one developed carious lesion (stage 3 or 4 according to the Eckstrand classification for carious lesions) either on a deciduous or a permanent tooth [37]
		or	At least one infected lesion (presence of an abscess, or a tooth with pulpal exposure, or a fistula)
		or	Presence of complex orofacial dysmorphology
			**... regardless of the criteria that had a social impact**
**Undetermined**			At least one undetermined criteria that had a medical impact and regardless of the criteria that had a social impact.
Others conditions by elimination		or	Having no criteria that had a medical impact but having at least one undetermined criteria that had a social impact.

doi:10.1371/journal.pone.0002564.t002

COCNI Values	Suspected health conditions	CRITERIA
**3**<br>Urgent need for care or examination	Marker of local infectious disease	During the last three months the child expressed discomfort or pain in his/her mouth and consecutively he/she had no dental visit.<br>or At least one mucosal lesion on lips, tongue or jaw<br>or At least one infected lesion (presence of an abscess, or a tooth with pulp exposure cavity, or a fistula)<br>AND absence of any systemic disease* requiring specific oral health monitoring.
	Marker of focal infectious disease	During the last three months the child expressed discomfort or pain in his/her mouth and consecutively he/she had no dental visit.<br>or Presence of at least one mucosal lesion on lips, tongue or jaw<br>or At least one infected lesion (presence of an abscess, or a tooth with a pulp exposure, or a fistula)<br>or Presence of generalized gingivitis (Loe and Silness index >0 for both arches)<br>AND Existence of a systemic disease requiring specific oral health monitoring*
	Marker of traumatic lesions	During the last three months the child expressed discomfort or pain in his/her mouth and consecutively he/she had no dental visit.<br>or Presence of at least one mucosal lesion on lips, tongue or jaw
	Marker of oral disease with functional or social consequences	During the last three months the child expressed discomfort or pain with his/her mouth and consecutively he/she had no dental visit.
**2**<br>Need for care or examination	Marker of local infectious disease	Presence of calculus<br>or Presence of gingivitis<br>or Presence of at least one anterior tooth with a fractured, unrestored crown<br>or Presence of at least one developed carious lesion.<br>AND Absence of a systemic disease* requiring specific oral health monitoring
	Marker of focal infectious disease	Presence of calculus.<br>or Presence of a localized gingivitis<br>or Presence of at least one anterior tooth with a fractured, unrestored crown<br>or Presence of at least one developed carious lesion.<br>AND Existence of a systemic disease* requiring specific oral health monitoring
	Marker of traumatic lesions	Presence of at least one anterior tooth with a fractured, unrestored crown
**1**<br>Need for examination	Marker of local infectious disease	No dental visit over the last 12 months<br>or Presence of dental plaque<br>or Presence of at least one incipient carious lesion.<br>AND Absence of a systemic disease* requiring specific oral health monitoring
	Marker of focal infectious disease	No dental visit over the last 12 months<br>or Presence of dental plaque<br>or Presence of at least one incipient carious lesion.<br>AND Existence of a systemic disease* requiring specific oral health monitoring
	Marker of traumatic lesions	No dental visit over the last 12 months
	Marker of oral disease with functional or social consequences	No dental visit over the last 12 months<br>or Presence of an anterior edentulous segment from 1 to 6 teeth on at least one arch.<br>or Presence of a posterior edentulous segment for children up to 13 years old)<br>or Presence of untreated severe orofacial dysmorphology
**0**<br>No need for care nor examination		Other conditions by elimination

*Epilepsy, congenital cardiac disease, bronchopneumopathy (including asthma), internal prosthesis, immunodeficiency and hematological disease, or diabetes.

doi:10.1371/journal.pone.0002564.t003

COPI Values		CRITERIA
**1**		Existence of systemic disease requiring specific oral health monitoring*
Existence of at least one preventive or dental health education action need	or	Presence of dental plaque
	or	Lack of autonomy for feeding
	or	Being fed by tube or parenteral nutrition
	or	Restriction to puréed foods
	or	Eating hyper-calorific food complements or drinking sweetened drinks
	or	Coughing regularly during meals
	or	Presence of halitosis
	or	Having difficulties communicating pain
	or	Drooling
	or	Being uncooperative during oral examination [38]
**0**		
No need for either preventive health action or dental education		Other conditions, by elimination

*as defined in the descriptive results section.

doi:10.1371/journal.pone.0002564.t004
