# Supplementary material for: Oral health-related quality of life of Portuguese adults with mild intellectual disabilities
Source: PLoS One. 2018 Mar 21;13(3):e0193953. doi: 10.1371/journal.pone.0193953 (PMC5862473; doi:10.1371/journal.pone.0193953)
Supplement: S2 File — (PDF) [file pone.0193953.s002.pdf]

OHIP-14-MID-PT

Este questionário pergunta de que forma transtornos com os seus dentes, boca ou próteses podem causar-lhe problemas no seu dia-a-dia.

Responda às questões em baixo (colocando uma cruz atrás da opção que acha mais adequada) tendo em conta a frequência com que sentiu cada um dos aspetos nos **últimos 12 meses.**

1.Teve alguma dificuldade em pronunciar algumas palavras devido a problemas com os seus dentes, boca ou prótese dentária?

□Quase sempre □Muitas vezes □Às vezes □Quase nunca □Nunca □Não sei

2. Notou mais dificuldade em sentir o sabor dos alimentos devido a problemas com os seus dentes, boca ou prótese dentária?

□Quase sempre □Muitas vezes □Às vezes □Quase nunca □Nunca □Não sei

3. Teve alguma dor na sua boca?

□Quase sempre □Muitas vezes □Às vezes □Quase nunca □Nunca □Não sei

4. Sentiu algum desconforto quando come algum alimento devido a problemas com os seus dentes, boca ou prótese dentária?

□Quase sempre □Muitas vezes □Às vezes □Quase nunca □Nunca □Não sei

5. Tem-se sentido pouco à vontade por causa dos problemas com os seus dentes, boca ou prótese dentária?

□Quase sempre □Muitas vezes □Às vezes □Quase nunca □Nunca □Não sei

6. Sentiu-se nervoso(a) devido a problemas com os seus dentes, boca ou prótese dentária?

□Quase sempre □Muitas vezes □Às vezes □Quase nunca □Nunca □Não sei

7. Deixou de comer algum alimento devido a problemas com os seus dentes, boca ou prótese dentária?

□Quase sempre □Muitas vezes □Às vezes □Quase nunca □Nunca □Não sei

8. Teve de interromper refeições devido a problemas com os seus dentes, boca ou prótese dentária?

□Quase sempre □Muitas vezes □Às vezes □Quase nunca □Nunca □Não sei

9. Sentiu dificuldade em relaxar/descansar devido a problemas com os seus dentes, boca ou prótese dentária?

□Quase sempre □Muitas vezes □Às vezes □Quase nunca □Nunca □Não sei

10. Tem-se sentido um pouco envergonhado(a) devido a problemas com os seus dentes, boca ou prótese dentária?

□Quase sempre □Muitas vezes □Às vezes □Quase nunca □Nunca □Não sei

11. Tem sido menos tolerante ou paciente com o(a) seu(sua) companheiro(a) ou família devido a problemas com os seus dentes, boca ou prótese dentária?

□Quase sempre □Muitas vezes □Às vezes □Quase nunca □Nunca □Não sei

12. Teve dificuldade em realizar as suas atividades habituais por causa de problemas com os seus dentes, boca ou prótese dentária?

□Quase sempre □Muitas vezes □Às vezes □Quase nunca □Nunca □Não sei

13. Sentiu que a sua vida em geral tem corrido pior devido a problemas com os seus dentes, boca ou prótese dentária?

□Quase sempre □Muitas vezes □Às vezes □Quase nunca □Nunca □Não sei

14. Tem-se sentido completamente incapacitado devido a problemas com os seus dentes, boca ou prótese dentária?

□Quase sempre □Muitas vezes □Às vezes □Quase nunca □Nunca □Não sei
